# Supplementary material for: Content-rich biological network constructed by mining PubMed abstracts
Source: BMC Bioinformatics. 2004 Oct 8;5:147. doi: 10.1186/1471-2105-5-147 (PMC528731; doi:10.1186/1471-2105-5-147)
Supplement: Additional File 2 — The original results of the above study (non-essential files are deleted to keep the file size under the limit set by BMC bioinformatics). [file 1471-2105-5-147-S2.bz2 › chilibotAdditionalFile2/dip20/502ID7878020E10668/html/TGFB3_TGFBR2.html]

 


 **TGFB3** and **TGFBR2** 
  
Found 31 abstracts in PubMed, retrieved 20.  
 

 What does Google say? 
 PDF only 
| .edu only 

---

**Interactive relationship** (e.g. stimulation, inhibition, etc)

**Inhibitory relationship**- Whereas expression of TGF beta1, TGF beta2, and TGF beta type II receptor  [ **TGFBR2** ]  was suppressed by glucocorticoids in normal and wounded skin, expression of TGF beta3  [ **TGFB3** ]  and TGF beta receptor type I mRNA was stimulated.  Ref: 8626581 J Biol Chem, 1996
**Neutral relationship**- Furthermore, competition based quantitative reverse transcription PCR using synthetic internal standards revealed that leiomyomata express a significantly higher number copies per cell of TGF beta type II receptor  [ **TGFBR2** ]  mRNA, followed by TGF beta 1, TGF beta type I receptor, TGF beta 2, and TGF beta 3  [ **TGFB3** ]  P <.  Ref: 8784073 J Clin Endocrinol Metab, 1996

**Non-interactive relationship** (e.g. studied together, co-existance, homology, etc.)

- In situ analysis of transforming growth factors beta TGF beta 1, TGF beta 2, TGF beta 3  [ **TGFB3** ]  and TGF beta type II receptor  [ **TGFBR2** ]  expression in basal cell carcinomas.  Ref: 8763422 Br J Dermatol, 1996
- Immunohistochemical localization of TGF beta type II receptor  [ **TGFBR2** ]  and TGF beta3  [ **TGFB3** ]  during palatogenesis in vivo and in vitro.  Ref: 9727838 Int J Dev Biol, 1998
- Messenger RNA was isolated from the nucleus pulposus using oligo 25 superparamagnetic beads and probing with gene specific primers in RT PCR. mRNA coding for TGF alpha, EGF, TGF beta1 and TGF beta3  [ **TGFB3** ]  or the EGF receptor and TGF beta type II receptor  [ **TGFBR2** ]  was found only occasionally.  Ref: 10615986 J Bone Joint Surg Br, 1999
- We therefore studied the expression of UPA, UPAR, IGF2R, ALK5,  **TGFBR2** , TGFBR3, ENG, ALK1, TGFB1, TGFB2, and  **TGFB3**  in a series of 14 pancreatic carcinoma cell lines.  Ref: 11408925 Int J Oncol, 2001
- In the present study, we have analysed and compared, by in situ hybridization, mRNA expression of transforming growth factors beta TGF beta 1, TGF beta 2, TGF beta 3  [ **TGFB3** ]  and TGF beta type II receptor  [ **TGFBR2** ]  T beta R II, and, by immunohistochemistry, the distribution of TGF beta 3  [ **TGFB3** ]  protein in normal human skin and in basal cell carcinoma BCC.  Ref: 8763422 Br J Dermatol, 1996
- In this study we have investigated the expression of the precursor forms of TGF beta1, TGF beta2, TGF beta3  [ **TGFB3** ] , as well as TGF beta type I receptor TGF betaRI and TGF beta type II receptor  [ **TGFBR2** ]  TGF betaRII in the synovial tissue of arthritic rats during the course of the disease.  Ref: 9010265 Clin Exp Immunol, 1997
- Here, we present the 2.15 A crystal structure of the extracellular ligand binding domain of the human TGF beta type II receptor  [ **TGFBR2** ]  ecTbetaR2 in complex with human TGF beta3  [ **TGFB3** ] .  Ref: 11850637 Nat Struct Biol, 2002
- Corresponding increases in the levels of proliferating cell nuclear antigen PCNA and intracellular TGF beta 3  [ **TGFB3** ]  expression and a downregulation of the TGF beta type II receptor  [ **TGFBR2** ]  expression were also observed in the granulation tissue of the ulcer margins.  Ref: 9361170 Scand J Gastroenterol, 1997
- In this assay, the apparent dissociation constant of the TGF beta type II receptor  [ **TGFBR2** ]  ectodomain for TGF beta 3  [ **TGFB3** ]  was approximately 150 nM this value is approximately 1000 fold higher than that of the cell membrane receptor complex of living cells.  Ref: 8917430 Eur J Biochem, 1996
